# Examining the effect of threaded bolt fasteners on steel construction

**DOI:** 10.1038/s41598-024-67428-5

**Published:** 2024-07-16

**Authors:** Mehmet E. Uz, Emre Ozkat, Mehmet C. Ersoy, Necdet Salvan

**Affiliations:** Aydin Adnan Menderes University, 09100 Aydin, Turkey

**Keywords:** Shear out, Shear-bolted connection, Threaded bolts, Finite element analysis, Civil engineering, Metals and alloys

## Abstract

This study investigated the effects of threaded bolt fasteners on the initial and final stiffness of bolted joints in special steel modular construction. A comprehensive set of 246 specimens, including 4, 7.6, and 12 mm thick plates with 20 and 30 mm diameter bolts and different end spacings, were tested. The results revealed that connections with threaded bolts exhibited lower initial stiffness compared to pure shaft connections. This study introduced a novel formula that significantly improved simulation results for bolted joints, surpassing previous modeling approaches. Following the American Society for Testing and Materials (ASTM) definition, the “elastic” stiffness values obtained from the laboratory tests were compared with those of Eurocode provisions. Additionally, ad hoc formulas were proposed for the stiffness of shank, lap connection with partially threaded, and fully threaded bolts. These results offer valuable insights for enhancing the stiffness of bolted shear connections.

## Introduction

Several researchers have experimentally and numerically investigated bolted shear connections^1–10^. However, to the best of our knowledge, none of these finite element (FE) analyses accounted for the presence of bolt threads. Specific studies^2,5,6,9^ exclusively utilized shank-bolted connectors. Može and Piculin^9^ also demonstrate a comparison between the current Eurocode linear load-deformation model for bearings. Their work illustrates that the proposed load-deformation relationship for bearings at bolt holes in this study, now incorporated in the latest Eurocode version for steel structure joint design, accurately predicts load-deformation behavior.

Conversely, researchers who conducted laboratory experiments with threaded bolts^3,4,7,8^ observed significant variations in initial stiffness between their test samples and FE models. Latour and Rizzano^8^ examined specific local impacts, including how the threads cause bending in both the bolt and the hole. Lim and Nethercot^7^ addressed this by incorporating the disparity between the major and minor diameters of the bolts when establishing the initial FE curve, resulting in satisfactory agreement in the middle of the overall response. Additionally, D'Antimo, Demonceau, Jaspart, Latour and Rizzano^4^ attributed the greater flexibility observed in their experimental results primarily to geometric imperfections surrounding the bolt holes. To match the experimental initial stiffness to their simulations, they intentionally reduced the bolt diameter. However, this approach significantly underestimated its ultimate strength. In both cases^4,7^ (as explained later), noticeable disparities were observed between the modified FE and experimental curves.

This study aimed to analyze the results of laboratory tests conducted on 123 specimens having different types of bolted connections (threaded, partially threaded, and full shank) to investigate the effect of threads on the flexibility of a shear connection. In some studies^2^ that did not consider threads, numerical model predictions exhibited considerably higher stiffness compared to experimental data. This study aimed to compare laboratory findings with predictions made by Ahmed and Teh^11^, the Eurocode (ECS^12,13^), and Rex and Easterling^3^. Furthermore, ad hoc formulas were proposed to estimate rigidity for shank- and thread-bolted connections.

This study aims to develop FE models capable of accurately replicating the experimental behavior of shank- and thread-bolted connections. FE models are compared with previous studies^2,4,7^ to validate their accuracy. The objective was to utilize FE analysis results to explain the greater flexibility observed in experimental responses. Furthermore, the analysis helped elucidate the “stiffening” phenomenon exhibited by thread-bolted connections as they approach their final limit state. Notably, Haidar, Obeed and Jawad^14^; Hu, Shen, Nie, Yang and Sha^15^; and Zhang, Gao and Xu^16^ incorporated bolt threads into their FE analyses. However, their studies focused on bolted connections subjected to tension and the interaction between bolts and nuts. In contrast, this study focuses on the behavior of bolted shear connections, which has received limited attention in previous studies. Consequently, our research concentrates on simulating the load–displacement response of a bolted shear connection until reaching its ultimate limit load. Hence, the post-final load–displacement path is beyond the scope of this investigation.

## Configuration and specimen setup

**Figure 1:**
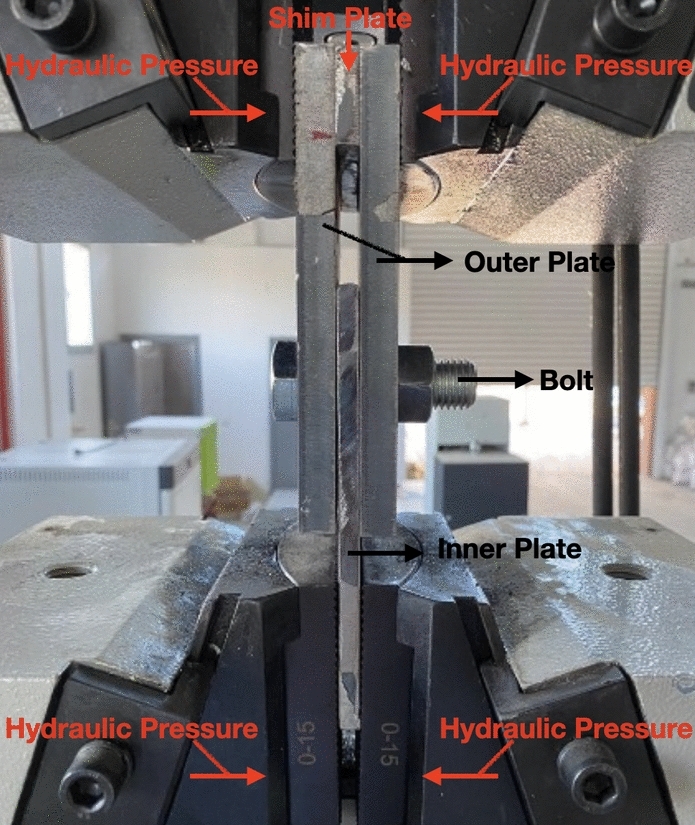
Specimen setup in laboratory.

This experiment examined three configurations of steel plates: 4, 7.6, and 12 mm thick. The 4 mm plates had a yield stress ${F}_{y}$ of 360 MPa and a tensile strength ${F}_{u}$ of 430 MPa. For the 7.6 mm plates, a yield stress of 340 MPa and a tensile strength of 488 MPa were measured, while the 12 mm plates exhibited a tensile strength of 520 MPa and a yield stress of 340 MPa. Partially and fully threaded shank bolts with nominal diameters of 20 and 30 mm were utilized in this study. Most plates had a width of 100 mm (*w* = 100 mm), but some were 50, 80, and 130 mm wide. A single-bolted double-shear connection was employed, where manual tightening of the bolt head and nut was conducted, as shown in Fig. 1. The inner plate, with a thickness of 12 mm and a yield stress of 400 MPa, played a crucial role in supporting the applied load. Due to its thickness, yield stress, and encountering only half the applied force compared to the outer plates, the distortion of bolt holes in the inner plate was insignificant. A BMT-600 s servo-hydraulic universal tensile testing machine was used to induce shear failure of a single-bolted joint caused by bolt-hole deformation. Shear-out failure predominated as the failure mode. Specimens were loaded at a stroke rate of 2 mm per min. Displacement is measured from the crosshead of Besmak Tension machine. Considering the inner plate as statically facing the applied force directly, the crosshead displacement of the entire system can effectively be attributed to the inner plate for force equilibrium. Additionally, clearances of 1 and 2 mm were allotted to the 20 and 30 mm bolts, respectively.

**Figure 2:**
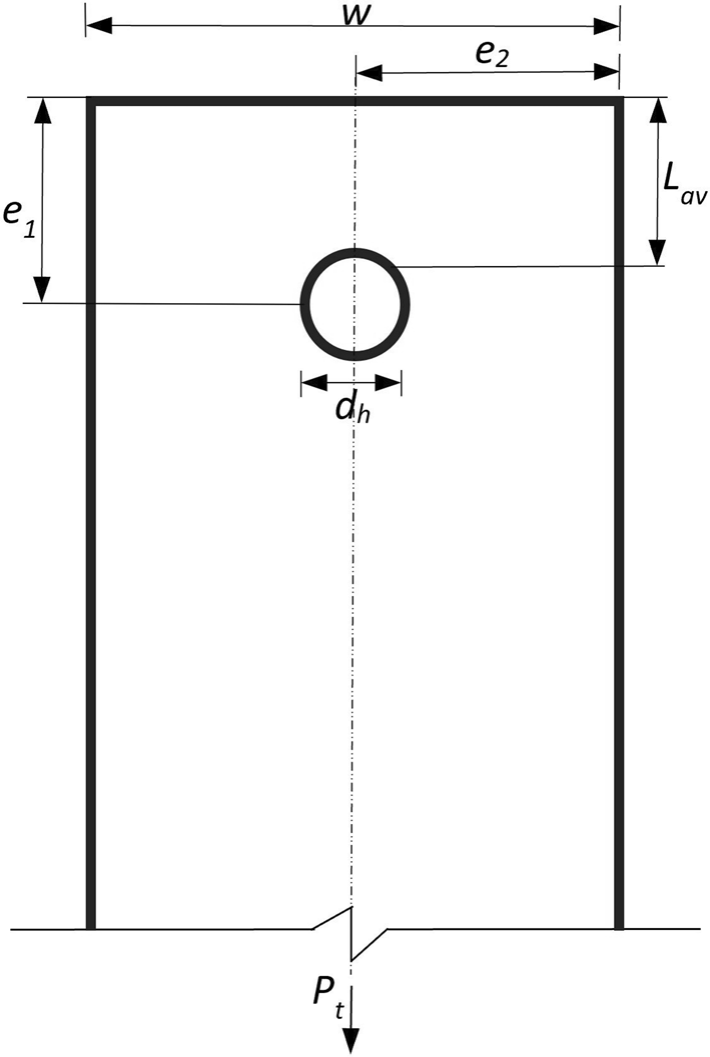
Dimensions of bolted connections.

Figure 2 illustrates the geometric features of the lab specimens. Tables 1, 2 and 3 contain data on plate thickness $t$, bolt hole diameter ${d}_{h}$, end distance ${e}_{1}$, plate width $w$, and bolt diameter $d$. Specimens were categorized as follows: BS for full-shank bolts, BT for threaded bolts, and BST for partially threaded bolts in this study.

**Table 1:** Dimensions and test results for threaded bolted specimens compared with the predicted values.

Specimen	$$F_{y} $$(MPa)	$$F_{u}$$(MPa)	$$d$$ (mm)	$$d_{h}$$(mm)	$$e_{1}$$(mm)	$$t$$(mm)	$$w$$(mm)	$$P_{t} /P_{p}$$	$$S_{t} /S_{eu}$$	$$S_{t} /S_{re}$$	$$S_{t} /S_{at}$$	$$S_{t} /S_{p}$$
BT-50	470	540	20	21.0	30.0	3.93	50	0.83	1.40	0.11	0.73	1.28
BT-44				21.0	30.0	3.89	80	0.92	1.32	0.11	0.68	1.21
BT-45				21.0	39.9	3.97		0.87	1.18	0.09	0.61	1.08
BT-46				21.0	49.8	3.98		0.82	1.14	0.09	0.59	1.04
BT-47				21.0	59.8	3.90		0.72	1.13	0.09	0.59	1.04
BT-38				21.0	30.0	3.95	100	0.95	1.30	0.10	0.67	1.19
BT-39				20.9	40.1	3.99		0.87	1.48	0.12	0.77	1.35
BT-40				21.0	50.1	3.96		0.82	1.18	0.09	0.61	1.08
BT-41				21.0	60.0	3.96		0.75	1.04	0.08	0.54	0.95
BT-53				21.0	30.1	3.95	130	0.94	1.62	0.13	0.84	1.48
BT-54				21.0	40.2	3.99		0.88	1.44	0.11	0.74	1.31
BT-55				21.0	50.0	3.93		0.81	1.10	0.08	0.57	1.00
BT-56				21.0	59.9	3.92		0.76	1.11	0.08	0.58	1.02
BT-12	280	418		21.0	30.0	7.95	50	1.15	1.11	0.10	0.56	0.98
BT-18				21.0	29.9	7.91	80	1.22	1.09	0.10	0.55	0.96
BT-19				21.0	39.9	7.93		1.15	1.22	0.11	0.61	1.08
BT-20				20.9	49.8	7.91		1.07	1.22	0.11	0.61	1.07
BT-21				21.0	60.2	7.91		0.95	1.12	0.10	0.56	0.99
BT-30				21.0	30.0	7.90	100	1.34	1.17	0.10	0.58	1.03
BT-1				21.4	40.7	7.49		1.20	0.97	0.09	0.49	0.86
BT-8				20.8	51.7	7.48		1.13	0.98	0.09	0.49	0.86
BT-32				21.1	60.0	7.65		1.01	1.21	0.11	0.61	1.07
BT-24				21.0	30.1	7.89	130	1.26	1.11	0.10	0.56	0.98
BT-25				21.0	40.0	7.93		1.14	1.11	0.10	0.55	0.98
BT-26				21.0	50.0	7.85		1.05	1.16	0.10	0.58	1.02
BT-27				21.1	60.0	7.91		0.95	0.98	0.09	0.50	0.89
BT-62	470	540	30	32.0	50.1	3.96	80	0.82	0.65	0.06	0.45	0.85
BT-68				32.0	50.0	3.98	100	0.90	0.62	0.06	0.43	0.80
BT-69				32.0	60.0	3.98		0.84	0.67	0.06	0.46	0.87
BT-70				32.0	70.0	3.97		0.76	0.76	0.07	0.52	0.99
BT-74				31.9	49.9	3.92	130	0.89	0.64	0.06	0.44	0.84
BT-75				31.9	60.0	3.96		0.85	0.70	0.06	0.48	0.91
BT-76				32.0	70.1	3.98		0.82	0.68	0.06	0.47	0.88
BT-77				32.0	80.0	3.97		0.77	0.57	0.05	0.39	0.75
BT-80	280	418		32.0	50.0	7.94	80	1.09	0.71	0.07	0.48	0.90
BT-86				32.0	50.0	7.93	100	1.17	0.78	0.08	0.53	0.99
BT-87				31.9	59.9	7.94		1.08	0.74	0.07	0.50	0.93
BT-88				32.0	69.8	7.96		1.00	0.65	0.06	0.43	0.82
BT-92				32.0	49.9	7.99	130	1.13	0.66	0.07	0.44	0.83
BT-93				32.0	59.7	7.87		1.10	0.72	0.07	0.48	0.91
BT-94				32.0	69.9	7.95		1.03	0.74	0.07	0.50	0.94
Mean								0.97	1.00	0.09	0.55	1.00
COV								0.166	0.275	0.227	0.177	0.157

Empty table cells indicate that the value in the cell is same that of the cell that is directly above.

**Table 2:** Dimensions and test results for partially threaded bolted specimens compared with predicted values.

Specimen	$F_{y}$(MPa)	$F_{u}$(MPa)	$d$(mm)	$d_{h}$(mm)	$e_{1}$(mm)	$t$(mm)	$w$(mm)	$P_{t} /P_{p}$	$S_{t} /S_{eu}$	$S_{t} /S_{re}$	$S_{t} /S_{at}$	$S_{t} /S_{p}$
BST-21	470	540	20	21.0	30.1	3.99	50	0.84	1.24	0.10	0.79	1.04
BST-27				21.0	29.9	4.01	80	0.92	1.12	0.09	0.71	0.94
BST-28				21.0	39.9	4.03		0.86	1.26	0.10	0.80	1.05
BST-29				21.1	50.0	3.97		0.81	0.86	0.07	0.54	0.72
BST-30				21.0	59.9	4.02		0.74	0.99	0.08	0.63	0.83
BST-23				21.0	30.0	3.99	100	0.94	1.23	0.10	0.78	1.03
BST-24				21.0	39.9	4.02		0.86	1.45	0.11	0.92	1.22
BST-25				21.0	49.9	3.97		0.83	1.20	0.09	0.76	1.01
BST-26				20.9	59.9	3.98		0.75	1.14	0.09	0.72	0.96
BST-35				21.0	29.9	3.97	130	0.93	1.25	0.10	0.79	1.04
BST-36				21.0	39.9	3.98		0.87	1.27	0.10	0.80	1.06
BST-37				21.0	50.0	3.97		0.84	1.13	0.09	0.71	0.95
BST-38				21.0	59.9	3.98		0.77	1.14	0.09	0.72	0.95
BST-22	280	418		21.0	30.0	7.86	50	1.14	0.91	0.08	0.64	0.74
BST-31				21.0	29.9	7.88	80	1.25	0.82	0.07	0.57	0.66
BST-32				21.0	40.2	7.96		1.21	1.08	0.09	0.76	0.87
BST-33				21.1	49.7	7.93		1.08	1.20	0.10	0.84	0.97
BST-34				21.0	60.5	7.92		1.02	1.16	0.10	0.81	0.94
BST-17				21.0	29.9	7.79	100	1.29	1.19	0.11	0.83	0.97
BST-9				21.0	41.2	7.30		1.24	1.07	0.10	0.74	0.87
BST-12				21.0	51.3	7.42		1.15	0.93	0.08	0.65	0.76
BST-14				20.9	51.4	7.44		1.11	1.96	0.17	1.36	1.60
BST-39				21.1	29.9	7.98	130	1.31	0.99	0.09	0.70	0.80
BST-40				21.0	39.9	7.94		1.21	1.22	0.11	0.86	0.99
BST-41				21.0	49.8	7.89		1.11	1.29	0.11	0.91	1.05
BST-42				21.1	59.8	7.97		0.89	1.06	0.09	0.75	0.86
BST-49	470	540	30	32.0	50.0	3.98	80	0.80	0.81	0.07	0.69	0.97
BST-53				32.0	50.0	3.93	100	0.93	0.94	0.09	0.79	1.12
BST-54				32.0	59.9	3.94		0.89	0.85	0.08	0.73	1.02
BST-55				32.1	70.0	3.96		0.76	0.90	0.08	0.76	1.07
BST-63				32.0	49.9	3.94	130	0.89	0.95	0.09	0.80	1.13
BST-64				32.1	59.9	3.94		0.88	0.81	0.07	0.69	0.97
BST-65				32.1	69.9	3.93		0.86	0.77	0.07	0.66	0.93
BST-66				32.1	79.9	3.95		0.78	0.85	0.07	0.72	1.01
BST-45	280	418		32.1	49.7	7.82	80	1.17	0.91	0.09	0.87	1.07
BST-58				32.0	49.9	7.76	100	1.27	0.78	0.08	0.74	0.91
BST-59				32.0	60.0	7.70		1.18	0.83	0.08	0.78	0.97
BST-60				32.2	69.7	7.82		1.10	0.85	0.08	0.81	1.00
BST-69				32.1	49.7	7.95	130	1.23	0.86	0.09	0.82	1.01
BST-70				32.1	59.7	7.78		1.20	0.78	0.08	0.74	0.91
BST-71				32.1	70.0	7.82		1.08	0.95	0.09	0.91	1.12
Mean								1.00	1.05	0.09	0.77	0.98
COV								0.179	0.220	0.195	0.163	0.156

Empty table cells indicate that the value in the cell is same that of the cell that is directly above.

**Table 3:** Dimensions and test results for full-shank bolted specimens compared with predicted values.

Specimen	$F_{y}$(MPa)	$F_{u}$(MPa)	$d$(mm)	$d_{h}$(mm)	$e_{1}$(mm)	$t$(mm)	$w$(mm)	$P_{t} /P_{p}$	$S_{t} /S_{eu}$	$S_{t} /S_{re}$	$S_{t} /S_{at}$	$S_{t} /S_{p}$
BS-52	470	540	20	21.0	30.0	3.94	50	0.84	1.62	0.13	0.52	1.24
BS-46				21.0	29.9	3.90	80	0.92	1.59	0.13	0.51	1.22
BS-47				21.0	39.8	3.92		0.89	1.78	0.14	0.58	1.37
BS-48				21.0	49.9	3.96		0.78	1.64	0.13	0.53	1.26
BS-49				21.0	59.9	3.94		0.71	1.43	0.11	0.46	1.10
BS-40				21.0	30.1	3.98	100	0.96	1.38	0.11	0.45	1.06
BS-41				21.0	40.0	3.96		0.84	1.72	0.13	0.56	1.32
BS-42				21.0	50.0	3.98		0.85	1.57	0.12	0.51	1.20
BS-43				21.0	60.0	3.98		0.78	1.38	0.10	0.45	1.05
BS-55				21.0	30.0	3.95	130	0.99	2.01	0.16	0.65	1.54
BS-56				21.0	40.0	3.95		0.93	1.56	0.12	0.50	1.19
BS-57				21.0	50.0	3.96		0.87	1.55	0.12	0.50	1.19
BS-58				21.0	60.0	3.98		0.79	1.32	0.10	0.43	1.01
BS-22	280	418		21.0	30.0	7.91	50	1.12	1.13	0.10	0.35	0.85
BS-28				20.9	29.8	7.89	80	1.22	1.19	0.11	0.37	0.89
BS-29				21.0	40.0	7.83		1.12	1.29	0.11	0.41	0.97
BS-30				21.0	49.8	7.87		1.04	1.18	0.10	0.37	0.89
BS-31				21.0	60.0	7.98		0.93	1.20	0.10	0.38	0.90
BS-17				21.0	29.9	7.95	100	1.24	1.31	0.12	0.41	0.98
BS-1				20.8	41.1	7.41		1.19	1.26	0.11	0.40	0.95
BS-5				20.9	49.7	7.30		1.17	1.13	0.10	0.36	0.85
BS-19				21.0	59.9	7.87		1.02	1.38	0.12	0.43	1.04
BS-34				21.0	29.9	7.90	130	1.25	1.16	0.10	0.36	0.87
BS-35				21.0	40.0	7.96		1.15	1.41	0.12	0.44	1.06
BS-36				20.9	50.0	7.87		1.03	1.24	0.11	0.39	0.93
BS-37				21.0	59.9	7.77		1.05	1.13	0.10	0.35	0.84
BS-63	470	540	30	32.0	50.0	3.94	80	0.80	0.86	0.08	0.38	0.95
BS-69				32.0	50.0	3.96	100	0.89	0.83	0.08	0.37	0.92
BS-70				32.0	60.0	4.02		0.88	0.93	0.08	0.41	1.03
BS-71				32.0	69.9	3.98		0.77	0.94	0.08	0.42	1.04
BS-75				32.0	49.9	3.97	130	0.89	0.93	0.08	0.41	1.02
BS-76				31.9	60.0	4.01		0.87	0.85	0.08	0.38	0.94
BS-77				32.0	70.1	4.01		0.85	0.90	0.08	0.40	1.00
BS-78				32.0	80.2	3.95		0.80	0.87	0.08	0.39	0.96
BS-81	280	418		32.0	50.0	7.89	80	1.10	0.79	0.08	0.34	0.86
BS-87				32.0	50.0	7.91	100	1.19	0.81	0.08	0.35	0.88
BS-88				32.0	60.0	7.91		1.09	0.75	0.07	0.32	0.82
BS-89				32.1	69.9	7.95		0.98	0.80	0.08	0.35	0.88
BS-93				32.0	49.9	7.95	130	1.19	0.80	0.08	0.34	0.87
BS-94				32.0	59.9	7.93		1.12	0.95	0.09	0.41	1.03
BS-95				32.0	69.9	7.82		1.14	0.89	0.09	0.39	0.97
Mean								0.98	1.21	0.10	0.42	1.02
COV								0.158	0.269	0.204	0.176	0.159

Empty table cells indicate that the value in the cell is same that of the cell that is directly above.

## Results of laboratory tests and discussion

The equation proposed by Teh and Uz^17^ was utilized to predict specimens’ shear-out capabilities, as shown in Tables 1, 2 and 3. A professional factor was calculated by dividing the ultimate test load ${P}_{t}$ by the expected load ${P}_{p}$, which can be obtained using Eq. (1).

1 $$ P_{p} = 1.2L_{av} tF_{u} $$

where $L_{av}$ is the length of the active shear path as

2 $$ L_{av} = e_{1} - d_{h} /4 $$

**Figure 3:**
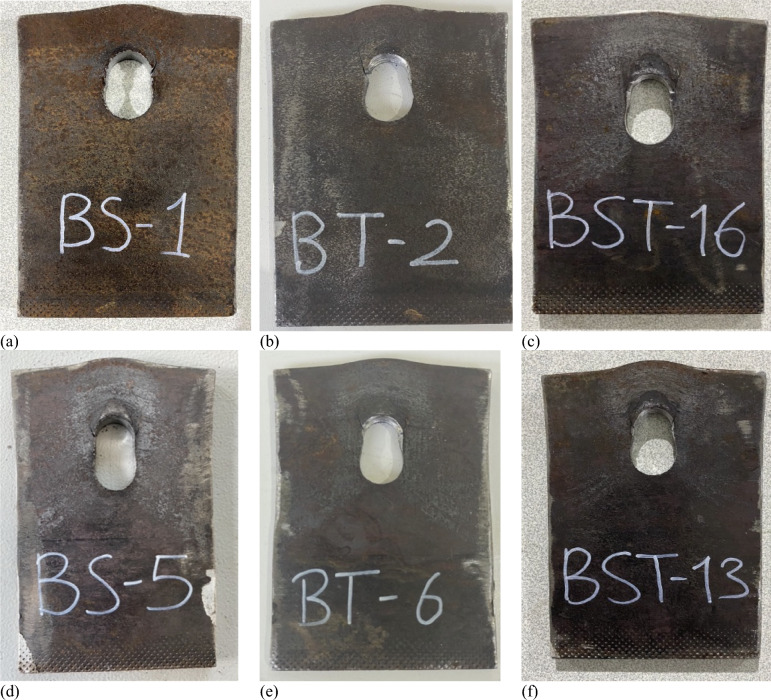
Shear-out failure mode: (**a**) BS1, (**b**) BS5, (**c**) BST16, (**d**) BT2, (**e**) BT6, and (**f**) BST13.

Figure 3 displays specimens tested in the lab for this study. BS1 and BS5 shared characteristics with BST13, BST16, BT2, and BT6, differing mainly in bolt thread variations. Both partially and fully threaded bolt holes showed damage, visible in accompanying photographs. Furthermore, a subtle difference in the elongation of bolt holes between shank and threaded specimens was present, along with a slight diameter variance between the straight shank portion and the tapered part of threaded holes, partially threaded holes falling between the two.

**Figure 4:**
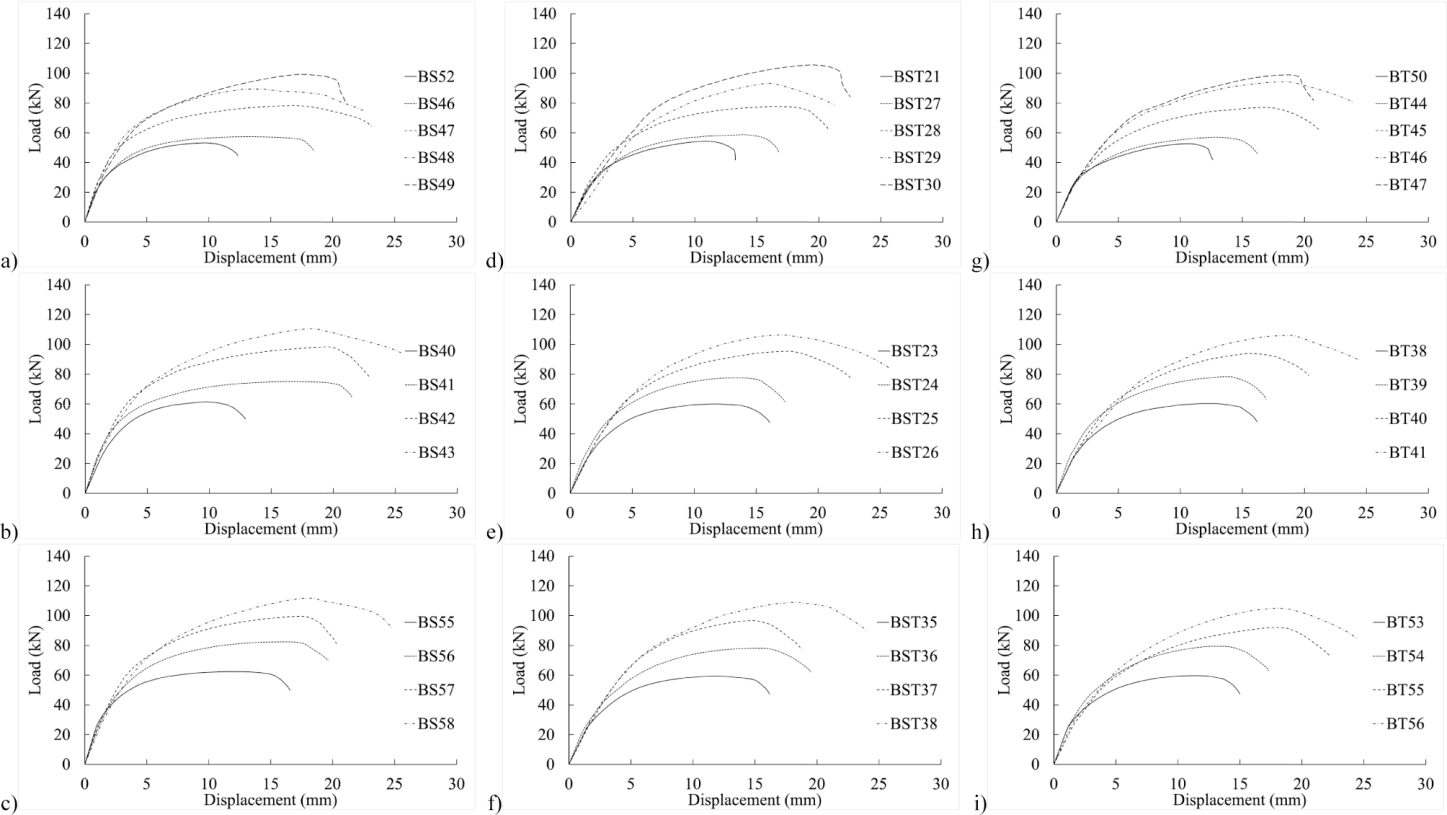
Experimental load–displacement graphs for 20 mm (**a**–**c**) full-shank, (**d**–**f**) partially threaded, and (**g**–**i**) threaded bolts in specimens of 4 mm thickness by changing the widths of 50, 80, and 100 mm.

Figure 4 exhibits load–displacement graphs for the 4 mm thick specimen. The initial stiffness of BS40–43 in shank-bolted shear connections appeared affected by the end distance ${e}_{1}$, as depicted in Fig. 4b, where increased end distance correlated with higher initial stiffness. This trend was similarly observed in partially threaded and threaded setups, as shown in Fig. 4e–h. Thus, variations in the end distance ${e}_{1}$ affected connection stiffness within practical bolted connection ranges.

Similarly, in the load–displacement graphs of threaded-bolt specimens, plate width and edge distance ${e}_{2}$ influenced the initial stiffness, indicating relative inconsistencies in specimen stiffness. Figures 4g–i illustrates that increased edge distances corresponded to decreased initial stiffness.

**Figure 5:**
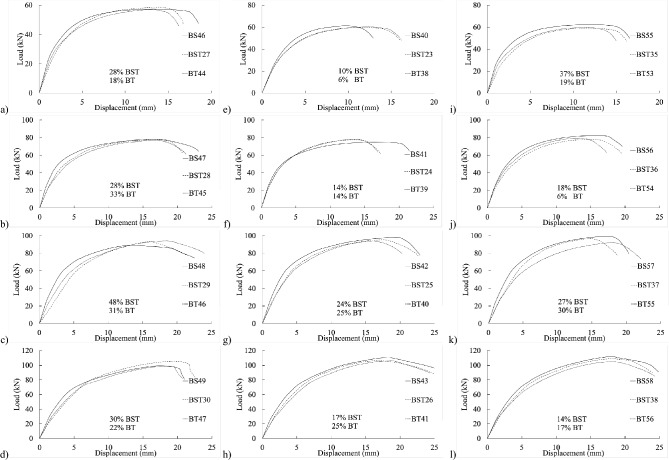
Load–displacement graph comparisons between 20 mm full-shank, partially threaded, and threaded bolts in specimens with a thickness of 4 mm and widths of (**a**–**d**) 80 mm, (**d**–**h**) 100 mm, and (**i**–**l**) 130 mm.

**Figure 6:**
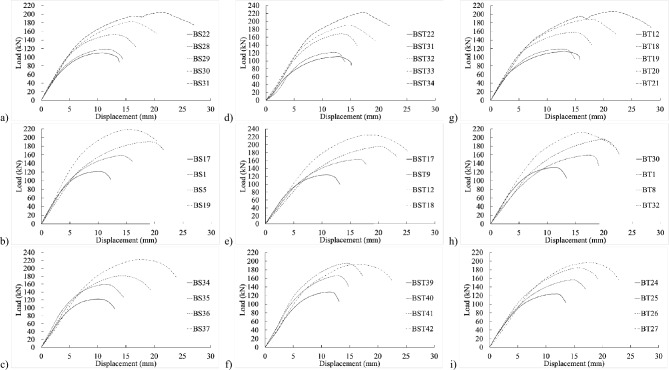
Experimental load–displacement graphs for 20 mm (**a**–**c**) full-shank, (**d**–**f**) partially threaded, and (**g**–**i**) threaded bolts in specimens with 8 mm thickness by changing the widths of 50, 80, and 100 mm.

**Figure 7:**
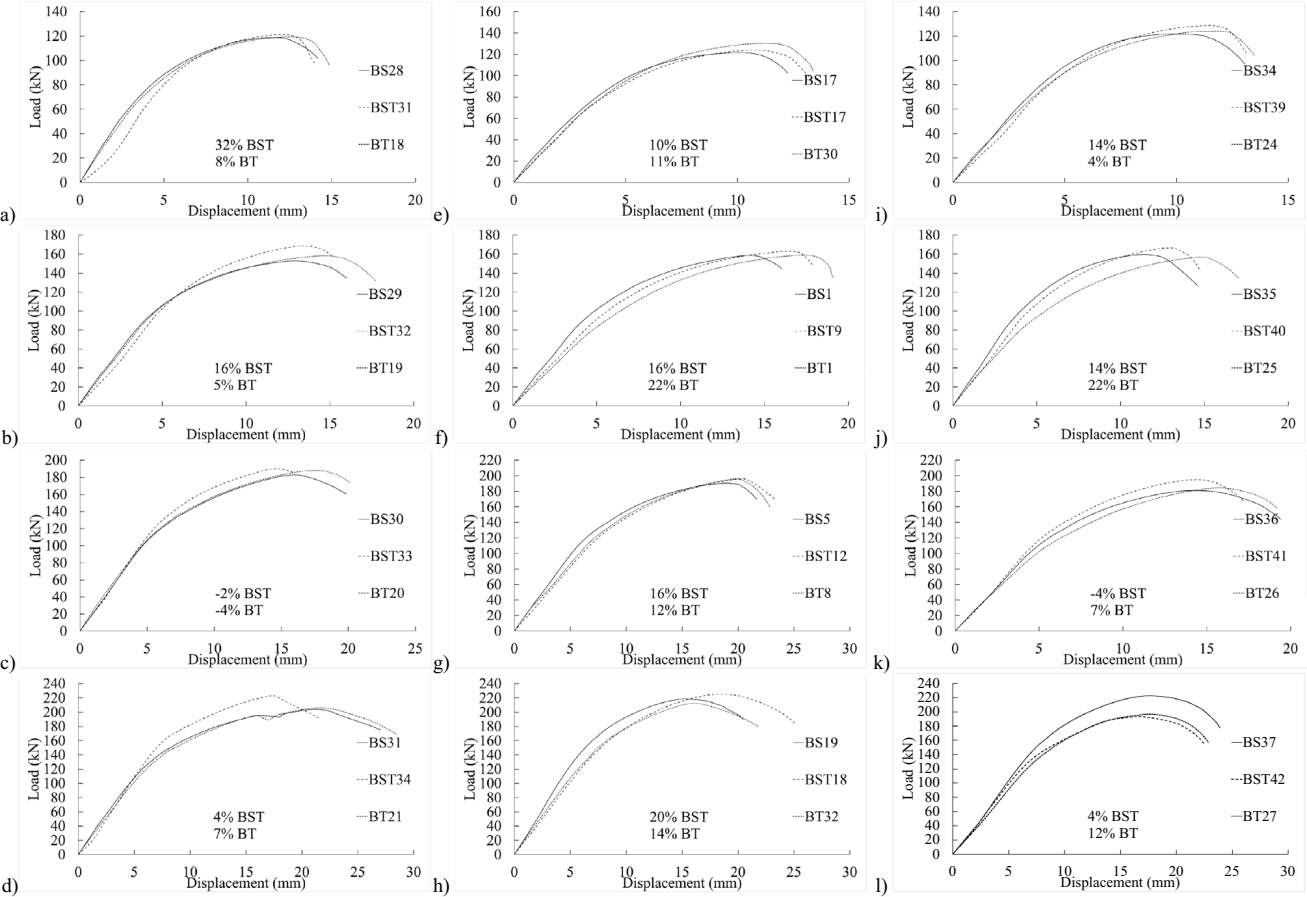
Load–displacement curve comparisons between 20 mm full-shank, partially threaded, and threaded bolts in specimens with thickness of 8 mm and widths of (**a**–**d**) 80 mm, (**d**–**h**) 100 mm, and (**i**–**l**) 130 mm.

Figure 5 displays load–displacement graphs of fully threaded, partially threaded, and shank-bolt specimens sharing similar traits within their respective categories. Specimen BT42 exhibited a 4% and 2% reduction in final shear-out capacity compared to specimens BS40 and BST25, respectively, sharing the same end distance ${e}_{1}$. Conversely, the threaded-bolt specimen exhibited a significantly lower initial stiffness than the shank-bolted one. A comparable trend was observed in the early rigidity of thicker specimens in Fig. 6b–d, albeit more pronounced. These trends are depicted in Fig. 7b,f,j, where increasing edge distances (*L*_*av*_) with constant end distances *e*_*1*_ resulted in a substantial decrease in initial stiffness. Similarly, Figs. 7a–c demonstrate a consistent pattern: increasing end distances while maintaining a constant edge distance.

**Figure 8:**
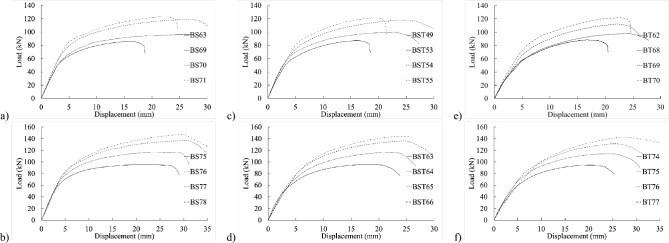
Experimental load–displacement graphs for 30 mm (**a**–**b**) full-shank, (**c**–**d**) partially threaded, and (**e**–**f**) threaded bolts in specimens with thickness of 4 mm by changing the widths to 80 and 100 mm.

**Figure 9:**
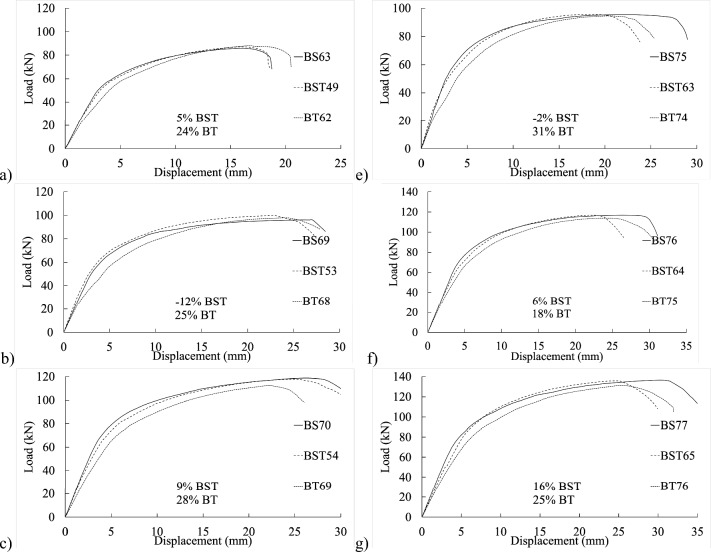

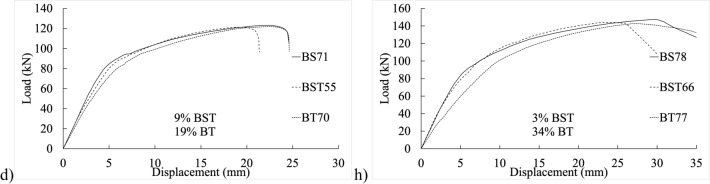
Load–displacement curve comparisons between 30 mm full-shank, partially threaded, and threaded bolts in specimens with a thickness of 4 mm and widths of (**a**–**d**) 100 mm and (**d**–**h**) 130 mm.

**Figure 10:**
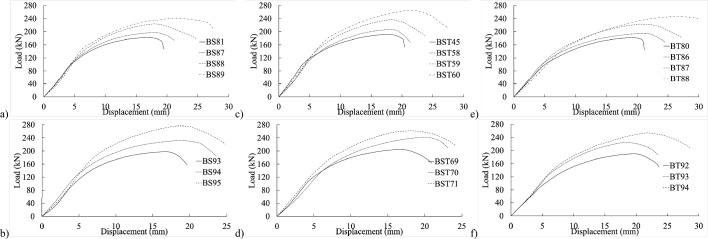
Experimental load–displacement graphs for 8 mm thick specimens with 30 mm (**a**–**b**) shank, (**c**–**d**) partially threaded, and (**e**–**f**) fully threaded bolts with widths of 80 and 100 mm.

**Figure 11:**
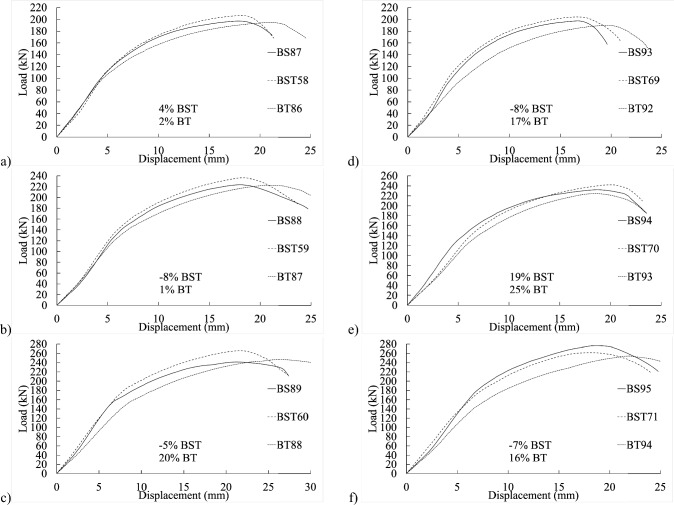
Load–displacement curve comparisons between 30 mm full shank, partially threaded, and threaded bolts in specimens with thickness of 8 mm and widths of (**a**–**c**) 100 mm and (**d**–**f**) 130 mm.

Notably, as threaded-bolt specimens approached their respective ultimate limit loads, they exhibited less softening behavior than shank-bolted specimens. When the bolt diameter was larger but the thickness was smaller, the initial stiffness followed a consistent pattern by adjusting edge and end distances (refer to Figs. 8 and 9). Conversely, as thickness and bolt diameter increased, the structure’s initial stiffness became more flexible (refer to Figs. 10 and 11). The latter part of this paper elaborates on how FE analysis was utilized to investigate this phenomenon. The study’s primary objective is to investigate the initial stiffness concerning the impact of thread or shanked bolts on bolt hole deformation.

## Verification of current equation

Section 9.1.3 of ASTM E2126-11^18^ defines the “elastic” stiffness ${S}_{t}$ for a bolted shear connection following the recommendations outlined in the standard:

3 $$ S_{t} = P_{0.4} /\Delta_{0.4} $$

where displacement ${\Delta }_{0.4}$ corresponds to the measured deflection under a load ${P}_{0.4}$, representing 40% of the ultimate test load ${P}_{t}$. Huth^19^ and the Eurocode (ECS^12^) present formulas for determining the elastic stiffness of bolted shear connections. Experimental results were compared with the Eurocode expression to evaluate the efficacy of structural steel connections.

## Eurocode 3

The formulas for two components of a double-shear bolted connection are outlined in Table 6.11 of Eurocode 3 (ECS^12^). To analyze a bolt in shear, the table provides the formula for the “elastic” stiffness, denoted as ${S}_{bs}$:

4 $$ S_{bs} = 8n_{b} d^{2} F_{ub} /d_{M16} $$

The term “elastic” stiffness ${S}_{pj}$, concerning a plate in bearing, is defined as a function of several variables. Specifically, ${d}_{M16}$ represents the specified size of an M16 bolt, ${n}_{b}$ denotes the number of bolt rows experiencing shear forces and the donation of ${n}_{b}$ in Eqs. (4) and (5) is taken in an account with two bolts per row in the new Eurocode^13^, and ${F}_{ub}$ denotes the tensile capacity of the bolt material. Eurocode^13^ specifies the stiffness coefficient for a bolt row consisting of two bolts. Therefore, the coefficient in Eqs. (4) and (5) are 8 and 12 and not 16 and 24 as in the Eurocode, respectively^9,10,20–22^. The initial model that predicts the load-deformation characteristics for bearings was already integrated into the inaugural iteration of the Eurocode. The ongoing research will take into account the new Eurocode^13^ for analyzing the bold embedment in the threaded shank bolt and partly bolt scenario.

5 $$ S_{pj} = 12n_{b} k_{b} k_{t} dF_{u} $$

where

6 $$ k_{b} = {\text{min}}\left( {k_{b1} ,k_{b2} } \right) $$

7 $$ k_{b1} = {\text{min}}(1.25,0.25e_{1} /d + 0.5) $$

8 $$ k_{b2} = {\text{min}}(1.25,0.25p/d + 0.375) $$

9 $$ k_{t} = {\text{min}}(2.5,1.5t/d_{M16} ) $$

As per Eurocode ECS^12^, evaluating the performance of a double-shear bolt connection hinges on specific characteristics—specifically, the downstream bolt’s end spacing (${e}_{1}$) and the bolt pitch $p$. These elements are regarded as a series of springs in the Eurocode’s assessment. In this double-shear bolt connection, three springs are in series, symbolizing the various stress types experienced by bolts and plates. The first spring represents the shear stress ${S}_{bs}$ on the bolt, the second covers the bearing stress ${S}_{po}$ on the two outer plates (operating in parallel), and the third accounts for the bearing stress ${S}_{pi}$ on the inner plate. The subscript “j” in ${S}_{pj}$ in Eq. (5) denotes either the outer “o” plate for ${S}_{po}$ or the inner “i” plate for ${S}_{pi}$, utilizing the related plate’s dimensions in Eqs. (6–9). The following equation calculates the equivalent spring stiffness ${S}_{e,eu}$ for the double-shear bolt connection:

10 $$ S_{e,eu} = \frac{1}{{\frac{1}{{S_{bs} }} + \frac{1}{{2S_{po} }} + \frac{1}{{S_{pi} }}}} $$

The Eurocode 3 (ECS^12^) does not explicitly distinguish between bolted connections with legs and those with threads. However, recent laboratory tests have revealed significant differences in the elasticity of bolted connections with a shank compared to those with a half-thread. Figure 11 illustrates this distinction. Tables 1, 2 and 3 depict the ratios between the measured elastic stiffness ${S}_{t}$ and the estimated equivalent spring stiffness ${S}_{e,eu}$. For bolted shank specimens, the average ratio was 1.21, with a coefficient of variation of 0.269, while for threaded bolted specimens, the corresponding values were 1.00 and 0.270, respectively. Notably, the definition of “elastic” stiffness derived from Eq. (3) may align with that used in the Eurocode’s provisions. This overscores the importance of carefully considering the implications of these results concerning Eurocode guidelines.

## Rex and Easterling^3^

Rex and Easterling^3^ proposed an alternative approach to estimate the initial stiffness of a single bolted connection. They defined initial stiffness as the stiffness at a displacement of 0.102 mm. Their analysis identified three key components contributing to initial stiffness: the bearing, bending, and shear stiffness of the plate, determined using Eqs. (11–14).

Bearing stiffness (Rex and Easterling^3^) is defined as:

11 $$ S_{br} = 120t\left( {\frac{d}{25.4}} \right)^{0.8} F_{y} $$

The following unit-dependent equation, with $d$ measured in millimeters, represents the plate bending stiffness as described by Rex and Easterling^3^:

12 $$ S_{b} = 32t\left( {\frac{{e_{1} }}{d} - \frac{1}{2}} \right)^{3} E $$

where Young’s modulus $E$ defines a plate’s stiffness, as demonstrated by Rex and Easterling^3^ in their study on plate shear stiffness, defined as

13 $$ S_{v} = 6.67t\left( {\frac{{e_{1} }}{d} - \frac{1}{2}} \right)G $$

The shear modulus of elasticity $G$ is utilized to determine the resultant connection stiffness $S_{e,re}$ by applying Eq. (14):

14 $$ S_{e,re} = \frac{1}{{\frac{1}{{S_{br} }} + \frac{1}{{S_{b} }} + \frac{1}{{S_{v} }}}} $$

The ratios of elastic stiffness ${S}_{t}$(measured according to ASTM E2126-11^18^) to the estimated ${S}_{e,re}$ are provided in Tables 1, 2 and 3. Shank-bolted samples showed an average ratio of 0.10, with a coefficient of variation of 0.204, whereas thread-bolted specimens had corresponding values of 0.09 and 0.227, respectively.

## Ahmed and Teh^11^

Ahmed and Teh^11^ considered the elastic stiffness of the tested connections as negligible, regardless of the end distance ${e}_{1}$. The authors also found that the connection movements were primarily a result of deformed bolt holes. The ad hoc formulas for shank and threaded-bolted connections, provided by Eqs. (15) and (16), respectively, are derived by incorporating the definition of elastic stiffness from Sect. 9.1.3 of ASTM E2126-11^18^ (expressed in Eq. (3)) and taking into account insights gained from laboratory test observations. The equations above assume a linear relationship between the connection stiffness and the number of bolts ${n}_{b}$, plate thickness $t$, and tensile strength of the material ${F}_{u}$.

To explore the correlation between bolt diameter *d* and stiffness, experimental tests are required, using M20 bolt specimens as a standard. The stiffnesses of the outer and inner plates, namely, ${S}_{eo}$ and ${S}_{ei}$, respectively, are calculated using Eqs. (15) and (16), in which the constants 24 and 15 in Eqs. (15) and (16), respectively, have been determined through empirical analysis of the illustrated test outcomes:

15 $$ S_{e,shank} = 24n_{b} t\left( {\frac{d}{{d_{M20} }}} \right)^{0.3} F_{u} $$

16 $$ S_{e,thread} = 15n_{b} t\left( {\frac{{d_{min} }}{{d_{min,M20} }}} \right)^{0.3} F_{u} $$

**Figure 12:**
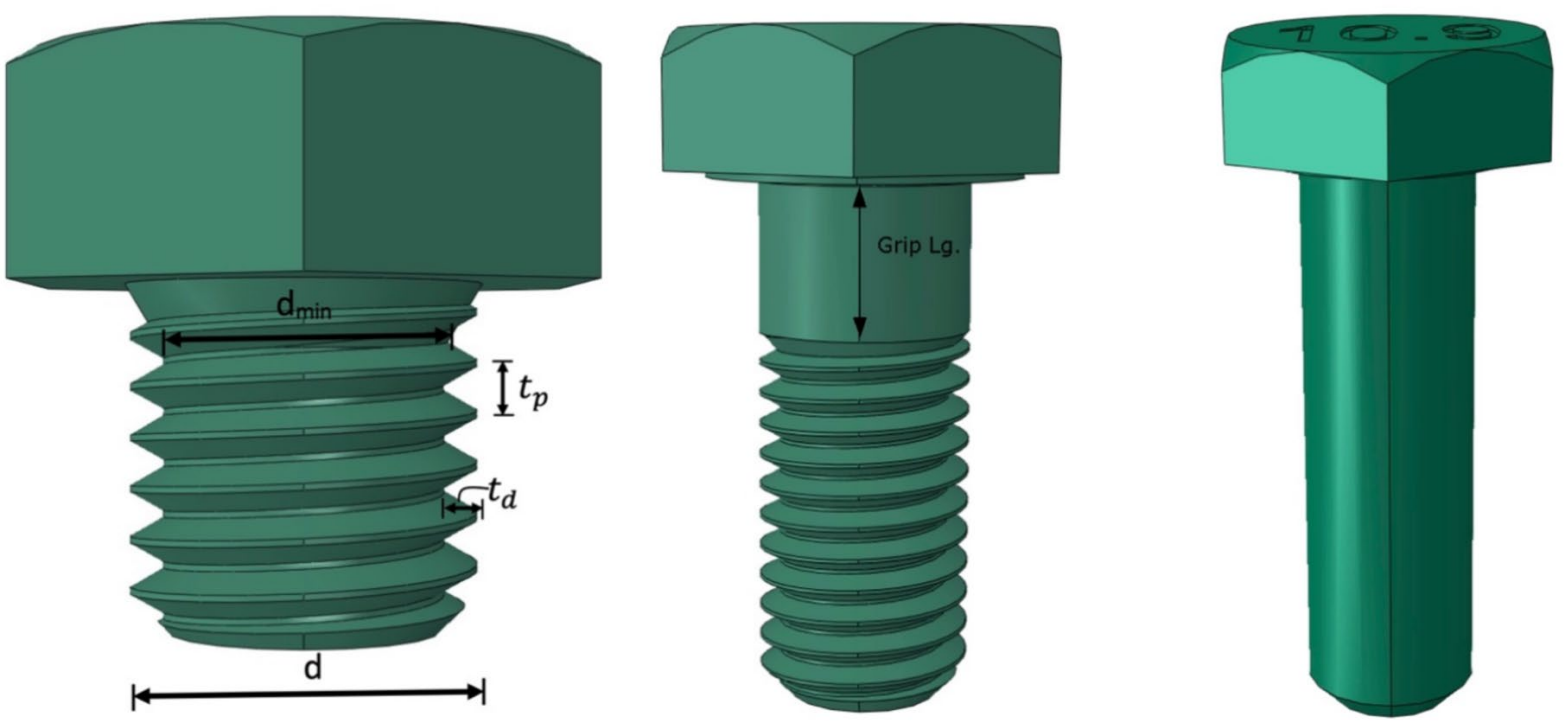
Dimensions of fully threaded (left), partially threaded (center), and shanked bolts (right).

Determining the combined stiffness of a dual shear-bolted joint relies on calculating the minor diameter ${d}_{min}$, obtained using the formula $d-2{t}_{d}$. The minor diameter ${d}_{min}$ and the thread depth ${t}_{d}$ are indicated in Fig. 12. The estimated stiffness for the assembly is calculated as follows:

17 $$ S_{e} = \frac{1}{{\frac{1}{{2S_{eo} }} + \frac{1}{{S_{ei} }}}} $$

Unlike Eq. (10), Eq. (17) disregards the bolt-shear component. The authors posit that according to the definition of elastic stiffness in Eq. (3), the bolt shear component is negligible compared to the bolt hole deformation. The final column in Tables 1, 2 and 3 presents the ratio of the measured stiffness ${S}_{t}$ to the estimated stiffness ${S}_{e}$. Shank-bolted specimens showed an average ratio of 0.42, with a coefficient of variation of 0.176. Thread-bolted specimens had corresponding values of 0.55 and 0.177, respectively.

## Proposed equation

The experimental findings clearly indicate that the gap between connection ends significantly affected their elastic stiffness. Additionally, the positioning of the bolt’s threads concerning the hole in the inner or outer plate exerted substantial influence on stiffness. Furthermore, bolt-hole deformation played a dominant role in joint displacement. Surprisingly, even the threading of one outer plate impacted the connection’s elastic stiffness. The elastic stiffness notably varied across different types of bolted connections, illustrated in Figs. 4, 5, 6, 7, 8, 9, 10 and 11. Depending on the bolt’s thread length, one outer plate faced the threaded side of the bolt while the other faced the shank. In a partially threaded connection, the upper outer plate’s hole interacted with the bolt’s shank. To establish the relationship between stiffness and bolt diameter, an empirical analysis was performed using M10 bolt specimens as a reference, as described in Eq. (18). Empirical values of 7, 9, and 1/6 were derived from the test results shown in Figs. 4, 5, 6, 7, 8, 9, 10 and 11. A linear relationship was assumed between stiffness and factors such as the number of fasteners, plate thickness, and material tensile strength.

18 $$ \begin{aligned} S_{e,thread - outer} = & 7n_{b} t_{outer} \left( {\frac{{d_{min} }}{{d_{min,M10} }}} \right)^{1/6} F_{u,outer} \\ S_{e,thread - inner} = & 7n_{b} t_{inner} \left( {\frac{{d_{min} }}{{d_{min,M10} }}} \right)^{1/6} F_{u,inner} \\ S_{e,shank - outer} = & 9n_{b} t_{outer} \left( {\frac{d}{{d_{M10} }}} \right)^{1/6} F_{u,outer} \\ S_{e,shank - inner} = & 9n_{b} t_{inner} \left( {\frac{d}{{d_{M10} }}} \right)^{1/6} F_{u,inner} \\ \end{aligned} $$

Typically, a double-shear bolted connection involves a series connection of three springs. However, as per Eq. (3), the definition of elastic stiffness suggests that the effect of bolt shear is minimal compared to the deformation in the bolt-hole^11,17^. Therefore, Eq. (19) presents a reasonable representation with two springs in series: one for the outer plates in parallel and another for the inner plate. Furthermore, Eq. (19) accounts for the interaction of the half-threaded and half-shank surfaces of the bolt in the inner plate, which interface with the bolt hole in parallel.

19 $$ \begin{aligned} S_{e,BT} = & \frac{1}{{\frac{1}{{2S_{e,thread - outer} }} + \frac{1}{{S_{e,thread - inner} }}}} \\ S_{e,BST} = & \frac{1}{{\frac{1}{{S_{e,thread - outer} + S_{e,shank - outer} }} + \frac{2}{{S_{e,thread - inner} + S_{e,shank - inner} }}}} \\ S_{e,BS} = & \frac{1}{{\frac{1}{{2S_{e,shank - outer} }} + \frac{1}{{S_{e,shank - inner} }}}} \\ \end{aligned} $$

**Figure 13:**
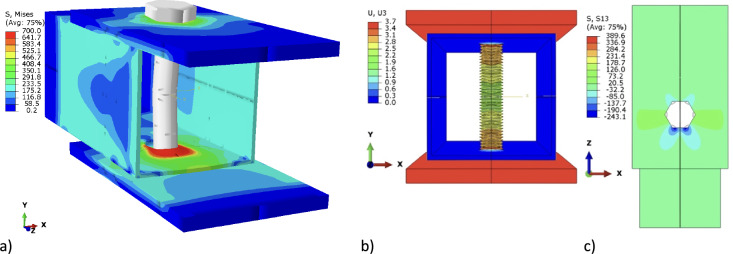
Result series HX-2-M12: (**a**) bolt failure (MPa) at the end of the test with local buckling in the FE model, (**b**) bolt elongation (mm) at an applied load of 59 kN, and (**c**) shear stress contours (MPa).

In accordance with the bolt length in Fig. 13, the outer and inner plates corresponded with the thread or shank in their respective bolt holes. The stiffness levels of the external and internal plates, ${S}_{e,outer}\text{ and }{S}_{e,inner},$ were calculated using Eq. (18). Specimens secured with a shank exhibited an average ratio of 1.02 with a coefficient of variation (COV) of 0.159. Threaded samples showed comparable values of 1.00 and 0.157, respectively.

## Finite element simulation

The plate and shank bolt were simulated using the hexahedral eight-node reduced integration brick element (C3D8R), while the threaded bolt employed the tetrahedral ten-node brick element (C3D10) in ABAQUS 6.14 standard software^23^. The software employed the Interaction module to replicate the precise movement of the bolt, especially when the internal plate faced loading. Steel material plasticity was incorporated using the von Mises yield criterion and the Prandtl–Reuss flow rule with isotropic hardening. Assumed material properties for this study included an elastic modulus of 200 GPa and a Poisson’s ratio of 0.30. To describe the stress–strain curve beyond the elastic region, the Ramberg–Osgood power model^24^ was applied, utilizing the corresponding material constants provided in Eqs. (20) and (21). This model is commonly used in engineering applications to accurately represent the nonlinear deformation of materials.

20 $$ \varepsilon = \frac{\sigma }{E} + 0.002\left( {\sigma /F_{y} } \right)^{{{\text{ln}}\left( {\varepsilon_{us} /0.2} \right)/{\text{ln}}(F_{u} /F_{y} )}} $$

21 $$ \varepsilon_{us} = 100\left[ {\varepsilon_{u} - \left( {F_{u} /E} \right)} \right] $$

where $\varepsilon$ denotes the engineering strain, and $\sigma$ denotes the engineering stress. The variable ${\varepsilon }_{u}$ is specifically defined as the ultimate stress-induced engineering strain, as indicated in Eq. (21). Having defining the engineering stress–strain relationship using Eqs. (20), the true stress–strain curve was plotted using Eqs. (22) and (23).

22 $$ \varepsilon_{True} = {\text{ln}}\left[ {1 + \varepsilon } \right] $$

23 $$ \sigma_{True} = \sigma \left[ {1 + \varepsilon } \right] $$

**Figure 14:**
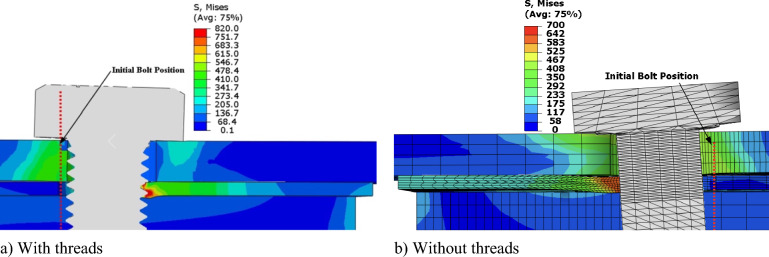
Deformed FE models of specimen HX-M12 under applied load of 32 kN.

**Figure 15:**
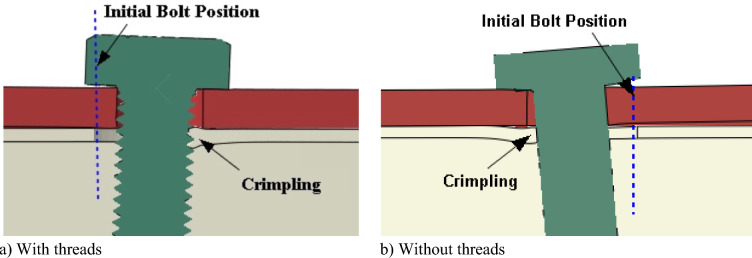
Deformed shape of specimen HX-M12 under applied load of 59 kN.

The symmetry control of a double-shear connection was achieved by explicitly modeling only one-fourth of the inner plate in ABAQUS, as depicted in Figs. 14 and 15, and applying appropriate boundary conditions. Movement along the *x*-axis was restricted for nodes on the in-plane symmetry plane, while nodes on the through-thickness symmetry plane were constrained from translating along the *y*-axis. As the C3D8R element used in this study did not require rotational degrees of freedom, they were omitted from the model. The bolt hole was discretized with a 1 mm mesh, following sensitivity analyses conducted by Ahmed and Teh^11^. Contact between components was modeled following Clements and Teh’s^25^ description. However, the existing models did not include the bolt threads, as elaborated later.

The ISO^26^ specifies the essential characteristics and thread specifications for fasteners with various diameters. Threads are generally classified as coarse, fine, or intermediate, depending on the fastener type. The provided equation explains the relationship between thread pitch ${t}_{p}$ and thread depth ${t}_{d}$:

24 $$ t_{d} = t_{p} \sqrt 3 /2 $$

In the Part section of ABAQUS^23^, a deformable 3D body with rotational and pitch characteristics represented the threaded bolt. In the Interaction section, node-to-surface discretization established surface contact. To accurately depict tangential behavior, a penalty formulation integrating friction coefficients was utilized, setting the values at 0.3 for threaded bolts and 0 for shank bolts.

## Shear connections utilizing bolts within a square hollow section

D’Antimo et al.’s study^4^ focused on double-shear bolt connections with square hollow sections (SHSs). Their experimental results demonstrated higher connection flexibility than predicted by their “ideal” FE models, which were based on the specified geometry of samples and the nominal bolt diameter. To improve alignment between experimental results and Finite Element analysis outcomes, the researchers used fastener models with reduced diameters to enhance initial stiffness. However, the simulated ultimate loads still exhibited an average decrease of 13% compared to the actual loads observed during experiments.

One specific specimen, HX-2-M-12 (Test 6)^4^, was analyzed using two different models: the first model accounted for bolt threads, while the second utilized the primary bolt diameter. In the second model, the bolt passed through a 2 mm-thick SHS with a 0.5 mm opening on each side. The 12 mm bolt had a coarse 1.75 mm thread pitch, and in this case, the end distance was 40 mm. An FE simulation was conducted considering the material properties and dimensions as measured by D’Antimo et al.^4^.

**Figure 16:**
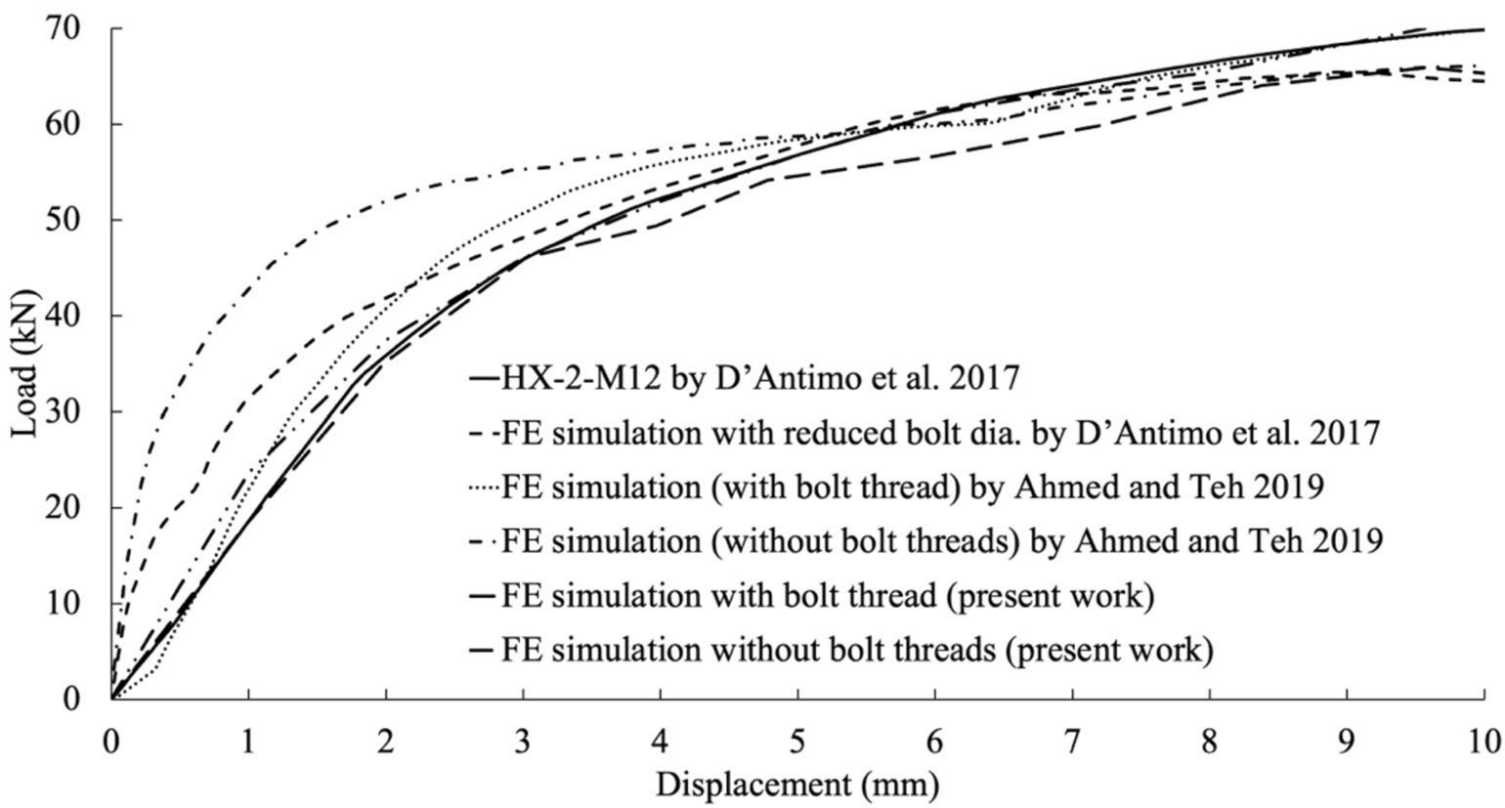
Comparison of load–displacement graphs of specimen HX-2-M12 obtained from the FE simulation in the current work with those used in studies conducted by other researchers.

The simulation employed a stress–strain curve determined by specific engineering parameters: a yield stress of 400 MPa, a tensile strength of 500 MPa, and a maximum stress elongation of 0.4. Both the threaded and shank-bolted models, depicted in Fig. 14, underwent a 30 kN load resulting in displacements of 1.3 mm and 0.4 mm, respectively. The presence of bolt threads caused them to intrude into the lateral surface of the SHS tube, decreasing the initial rigidity of the connection due to the cutting effect. Figure 15 displays the deformed shapes of the two FE models under a 59 kN load. The comparison of load–displacement graphs from laboratory tests and FE analyses in Fig. 16 shows that the model incorporating threaded bolts accurately replicated the experimental response, outperforming the model without threaded bolt representation. Interestingly, the model with bolt threads exhibited higher flexibility than the model without threads for loads up to approximately 55 kN, but beyond this point, the opposite behavior was observed. This trend aligned with the laboratory test results depicted in Figs. 5, 7, 9, and 11. Notably, the penetration of threads into the connected sidewall significantly reduced node overlap.

Figure 16 highlights the value of explicitly including bolt threads in this study. Load–displacement graphs from various FE models were compared with experimental test results. Specifically, the existing model, including the bolt’s thread, showed a stronger correlation with experimental data compared to the altered model suggested by D’Antimo et al.^4^, which employed a bolt with a smaller diameter. These findings underscore the significance of considering the impact of bolt threads when examining the behavior of double-shear bolt connections in structural investigations. Accurately representing threads significantly improves prediction accuracy, offering more reliable results.

## Lap connections incorporating double-shear

**Figure 17:**
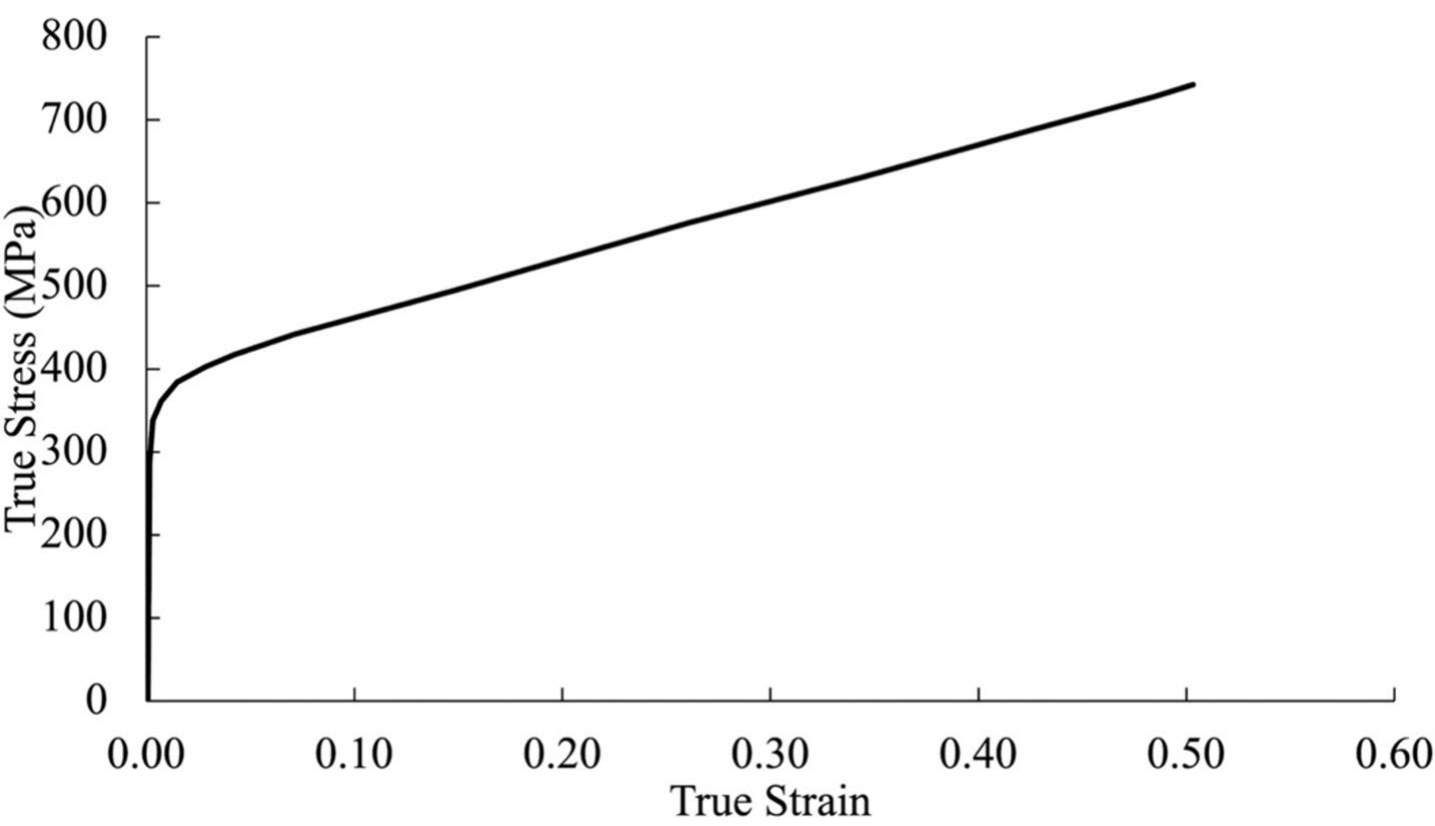
True stress–strain curve of 3 mm thick cold-formed steel tested by Lim and Nethercot^1^.

**Figure 18:**
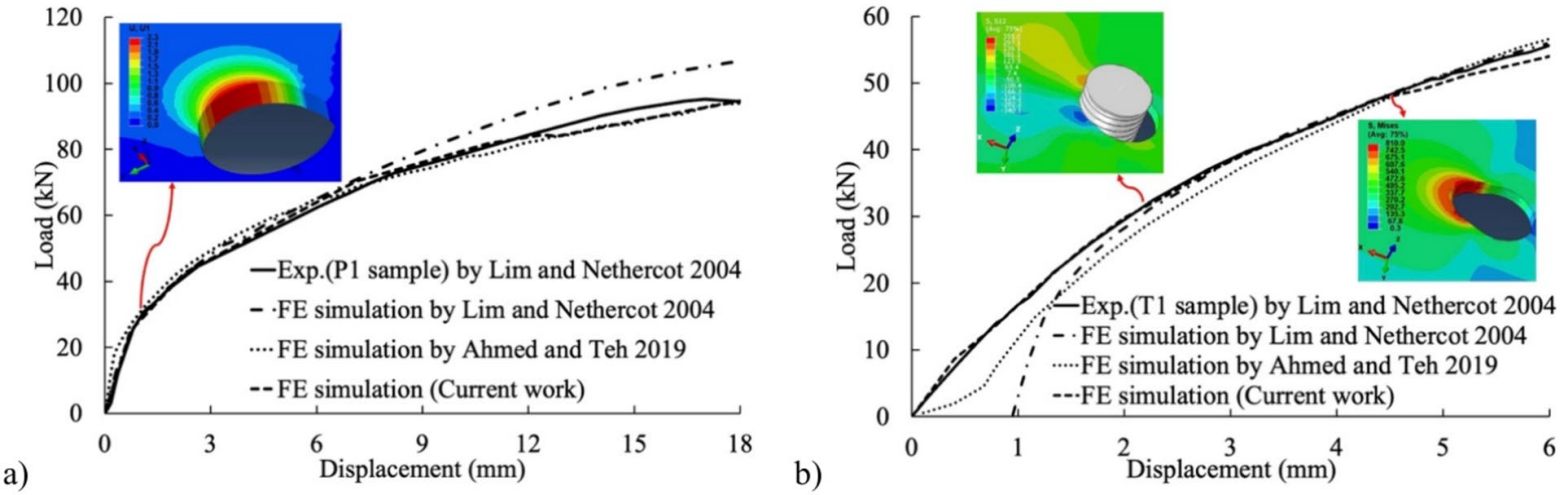
Comparisons between present FE results and experimental results of Lim and Nethercot^1^: (**a**) shank-bolted and (**b**) thread-bolted.

Lim and Nethercot^7^ examined double-shear lap connections featuring a single bolt, which could either be threaded or unthreaded. One specimen, labeled P1, comprised a 2.99 mm thick inner plate with a 17.7 mm bolt-hole diameter, connected using a shank bolt of 15.6 mm diameter. Conversely, P1’s threaded counterpart, denoted as T1, was 2.95 mm thick with a 17.8 mm bolt-hole diameter, associated with a bolt having a 15.8 mm major diameter and a 2 mm nominal coarse thread pitch. Both specimens maintained an 80 mm equal end distance. For accurate material representation, researchers utilized a true stress–strain curve (Fig. 17), showcasing engineering yield stress, tensile strength, and strain at the engineering maximum stress. To validate their FE models, researchers compared load–displacement curves from physical tests to those obtained from FE analyses of specimens fastened with shank bolts and threaded bolts, as depicted in Fig. 18a,b, respectively. It is crucial to point out that Ahmed and Teh^11^ implicitly assumed rigid and full constricted boundary conditions for the bolt, which do not accurately reflect experimental conditions. Notably, a strong agreement between experimental and simulated results in both cases underscores the reliability and accuracy of the models used in Lim and Nethercot’s study^7^, confirming the faithful depiction of the behavior of double-shear lap connections, regardless of shaft or threaded bolts.

## Stainless steel plate double-shear lap connections

**Figure 19:**
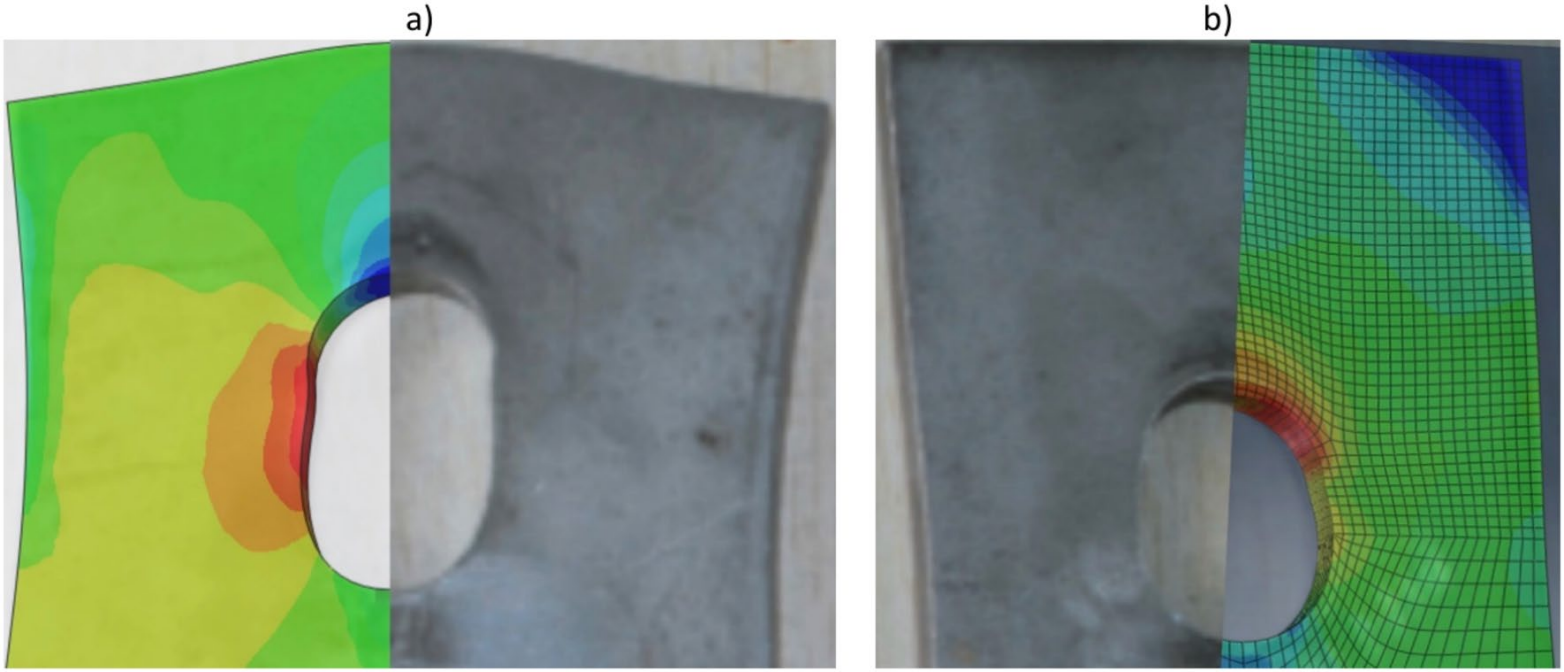
Comparison of failure modes between the FE analysis in the current study and test results from Yang et al.^2^: (**a**) S-2 and (**b**) S-5.

The double-shear bolted connections examined by Yang et al.^2^ involved 6 mm thick stainless steel plates connected to the shank portion of the bolts, without any threads. Despite the absence of thread influence, their FE load–displacement profiles exhibited significantly higher rigidity than their experimental joints, suggesting an alternative explanation for the more severe responses observed in the FE models. In our study, we aimed to replicate Yang et al.’s^2^ experiments by simulating specimens S-2 and S-5, featuring 20 and 24 mm bolt diameters, respectively. The bolt hole spacing was 2 mm, with specimen S-2 having a 40 mm end distance and specimen S-5 having a 60 mm end distance. Figure 19 illustrates the utilization of our current study’s FE models alongside Yang et al.’s^2^ experimental tests using these two distinct specimens.

**Figure 20:**
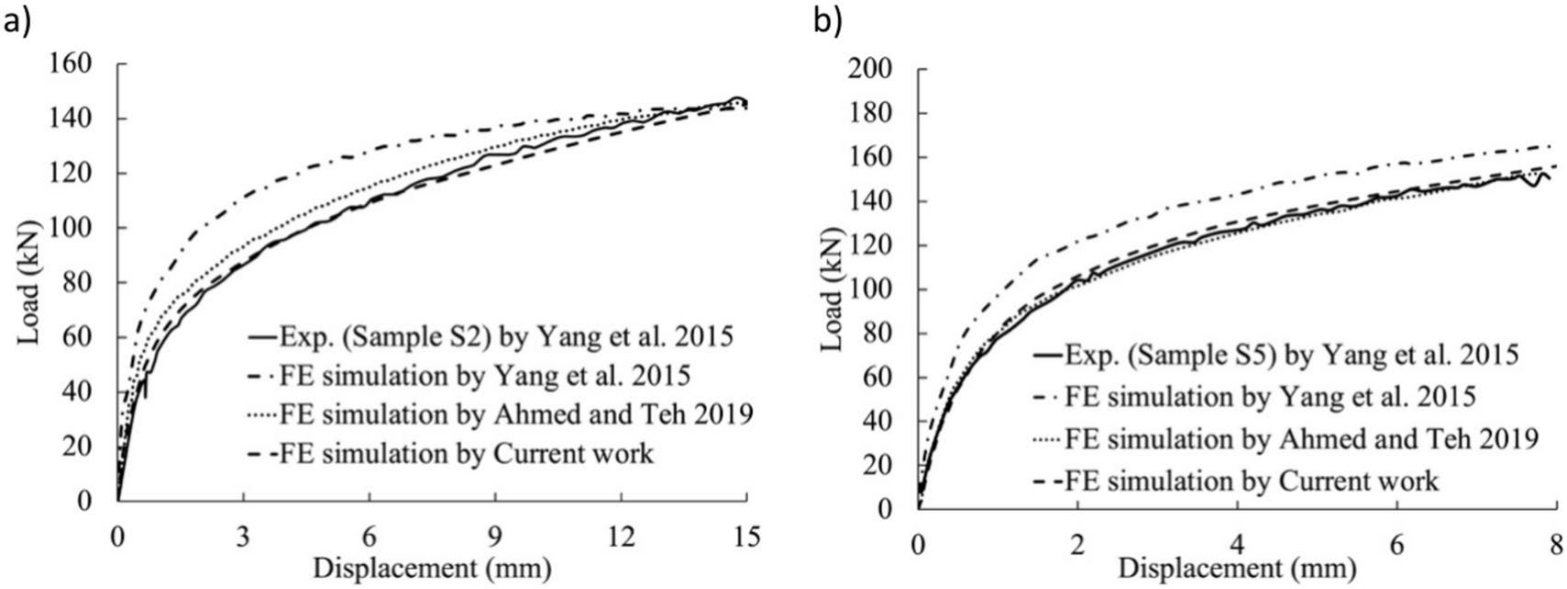
Comparison of FE load–displacement graphs for the specimens tested by Yang et al.^2^: (**a**) S-2 and (b) S-5.

Following Yang et al.’s findings^2^, this analysis employed the true stress–strain curve. Figure 20a,b present comparisons between load–displacement diagrams derived from laboratory experiments and those obtained by the authors and Yang et al.^2^ for the bolted shaft specimens S-2 and S-5, respectively. The experimental results were in good agreement with those of current models. The significant rigidity observed Yang et al.’s^2^ model could potentially be due to the coarser FE grid in their analyses, as depicted in one of the figures in their publication.

## Conclusion

This study examined the initial stiffness of fully threaded, partially threaded, and shank-bolted connections under shear loads using laboratory test data. Threaded specimens exhibited notably lower initial stiffness compared to shank specimens. FE calculations were crucial in understanding how bolt threads impacted shear stiffness. Results indicated a stiffness decrease due to threads, more pronounced in fully threaded bolts than partially threaded ones. The inclusion of bolt threads in FE analysis proved vital for accurately predicting the load–displacement response up to the ultimate stress point.

The paper presents findings from experiments and FE analyses of shear connections with threaded, half-threaded, and full-shank bolts. Threaded specimens displayed significantly lower initial rigidity than shank counterparts due to thread intrusion into plates. However, in bolted hollow sections, this intrusion decreased downstream plate deformation, resulting in increased threaded connection stiffness near ultimate loads. Edge and end distances minimally affected the elastic rigidity of threaded connections, predominantly influenced by bolt-hole deformation. The rigidity of each joint was mainly independent of bolt deformation for the same reason. Ad hoc formulations for half and full threaded and shank-bolted joint rigidity relied solely on bolt cavity deformation. Incorporating bolt threads in the FE model enabled accurate replication of load–displacement characteristics and effectively mirrored a separate specimen’s reaction under maximum load. This model outperformed a previous one using artificially reduced bolt characteristics, significantly underestimating the ultimate test load. However, these conclusions are specific to the findings of this study and might not universally apply to all bolted shear connections. Further research is essential to validate these conclusions and explore different connection behaviors under diverse conditions. Explicitly modeling threads proved superior to a model without threads, crucial for capturing the necessary initial rigidity in achieving accurate results. The ongoing study of the first author will analyze how the elongation of bolt holes affects the load deformation and elastic stiffness of bolt embedment involving threaded and shanked components.

## Data Availability

All data generated or analysed during this study are included in this published article.
